# Antidiabetic Properties of Germinated Brown Rice: A Systematic Review

**DOI:** 10.1155/2012/816501

**Published:** 2012-12-06

**Authors:** Mustapha Umar Imam, Nur Hanisah Azmi, Muhammad Iqbal Bhanger, Norsharina Ismail, Maznah Ismail

**Affiliations:** ^1^Laboratory of Molecular Biomedicine, Institute of Bioscience, Universiti Putra Malaysia, 43400 Serdang, Selangor, Malaysia; ^2^H.E.J. Research Institute of Chemistry, International Center for Chemical and Biological Sciences, University of Karachi, Karachi 75270, Pakistan; ^3^Department of Nutrition and Dietetics, Faculty of Medicine and Health Sciences, Universiti Putra Malaysia, 43400 Serdang, Selangor, Malaysia

## Abstract

Diet is an important variable in the course of type 2 diabetes, which has generated interest in dietary options like germinated brown rice (GBR) for effective management of the disease among rice-consuming populations. *In vitro* data and animal experiments show that GBR has potentials as a functional diet for managing this disease, and short-term clinical studies indicate encouraging results. Mechanisms for antidiabetic effects of GBR due to bioactive compounds like **γ**-aminobutyric acid (GABA), **γ**-oryzanol, dietary fibre, phenolics, vitamins, acylated steryl **β**-glucoside, and minerals include antihyperglycemia, low insulin index, antioxidative effect, antithrombosis, antihypertensive effect, hypocholesterolemia, and neuroprotective effects. The evidence so far suggests that there may be enormous benefits for diabetics in rice-consuming populations if white rice is replaced with GBR. However, long-term clinical studies are still needed to verify these findings on antidiabetic effects of GBR. Thus, we present a review on the antidiabetic properties of GBR from relevant preclinical and clinical studies, in order to provide detailed information on this subject for researchers to review the potential of GBR in combating this disease.

## 1. Introduction

Morbidity and mortality associated with type 2 diabetes is severe and significant, respectively. The statistics about the prevalence of this disease is very alarming as an estimated 346 million people were reported to be suffering from this disease in 2011 [1]. About 5% of all deaths globally each year are due to this disease, almost 80% of which occur in low- and middle-income countries [1, 2]. These figures are expected to grow especially in developing countries in near future. Rising incidence and prevalence of the disease points to factors that are still prevalent among vulnerable populations around the world. 

Complications arising from type 2 diabetes are debilitating and result in a huge expenditure in health care [1, 2]. The total cost of management of the disease, coupled with the accompanying economic loss due to lack of productivity, makes the search for remedies even more necessary. Also, the importance of dietary factors is acknowledged as a fundamental variable in the course of this disease [3–5]. This is very relevant for people who depend on foods that have been shown to increase the risk of and worsen type 2 diabetes. Foods with high glycemic index are reported to increase the risk of the disease and worsen it while, an opposite effect is indicated for those with low glycemic index [5, 6]. 

Implicated dietary, and other lifestyle factors in the pathogenesis of type 2 diabetes may be mediated at the epigenetic level, which is easily heritable [7]. The implication being the lifestyle choices of individuals could increase their risk of type 2 diabetes and the possibility of transmission of the same to off springs, even though they are devoid of genetic mutations. In the light of recent evidence therefore, lifestyle factors especially diet need to be given more attention in the management of type 2 diabetes. 

Drugs, dietary and lifestyle changes have been used in the management of type 2 diabetes. Despite advances in its management [8], incidence and prevalence of this disease is increasing [1, 2]. Failure to curb the rising incidence of the disease and its complications is indicative of inherent limitations of popular recommendations and already available treatment options, respectively. Drugs may have side effects in affecting compliance or may be out of reach in low- and middle-income countries due to financial constraints. Exercise may not fit the lifestyle of some individuals. Dietary modification may prove difficult for individuals especially when they cannot find suitable alternatives to what they commonly consume. An alternative option for reducing the risk of and/or managing type 2 diabetes may therefore have considerable impact on burden of the disease especially if it is practical for majority of people. 

For low- and middle-income countries, dietary management of type 2 diabetes should be given priority due to inaccessibility to drugs and other state-of-the-art facilities for managing the disease. Interestingly also, white rice (WR) is the staple food for majority of people in developing countries of Asia and Africa [9], where rise in incidence and prevalence of the disease is expected to be highest in near future [2]. In our opinion therefore, it is likely that WR may play some role in the growing incidence and prevalence of type 2 diabetes in these regions of the world since it has been linked to development of the disease [10]. So it is important to encourage less consumption of WR in these regions by providing a better alternative that will reduce the risk of the disease and likely promote health. This measure could significantly bring down the burden of this disease in those parts of the world. Limited option may be a reason why WR has a wide patronage around the world. Due to its high glycemic index [11], prolonged consumption may lead to disorders like obesity, glucose intolerance, type 2 diabetes, and cardiovascular diseases [12]. It may also worsen metabolic indices in type 2 diabetes.

In comparison to WR, there are many bioactives in brown rice (BR) with potential health benefits. Accordingly, Panlasigui and Thompson [13] reported that lower glycemic index as a result of BR consumption by healthy (12.1% lower) and diabetic subjects (35.6% lower) compared to WR could prevent type 2 diabetes and control glycemia, respectively. They linked these effects to the rich bioactive content of BR, which are removed in WR. Sun et al. [14] had shown, in a study involving over 200,000 subjects, that WR increased the risk of type 2 diabetes while replacing one-third of its daily serving with BR reduced the risk of developing type 2 diabetes. Following up almost 60,000 men and women for 5 years, Nanri et al. [15] also reported an increased risk of type 2 diabetes in women who consumed WR, but the association in men was less clear. Interestingly, Zhang et al. [16] reported that consuming WR instead of BR for 16 weeks in middle-aged Chinese men and women did not offer any benefits in terms of reduced risk of type 2 diabetes, though low population size could have resulted in lack of significant difference prompting them to recommend for more studies with larger sample size. In a meta-analysis and systematic review [10], however, involving more than 350,000 subjects followed up for 4–22 years, the risk of developing type 2 diabetes was clearly shown to be higher especially in Asians who consumed WR instead of BR. Furthermore, BR subjected to germination undergoes changes that significantly affect its properties, making it less chewy and richer in bioactives. These improve its functional effects [17]. In effect, WR is likely to increase risk of type 2 diabetes and worsen the disease in healthy and diabetic subjects, respectively, while BR and germinated brown rice (GBR) may offer benefits. This suggests that rice-consuming populations may derive nutritional and health benefits from BR and especially GBR more than WR. In this paper, we present findings from both clinical and preclinical studies on the promising role of GBR in management of type 2 diabetes.

## 2. Selection of Studies for Review

Using the electronic databases, Scopus, Google scholar, and Pubmed, a comprehensive and systematic search for publications was done without limiting the dates of publication. To identify the relevant literature in support of beneficial antidiabetic effects of GBR for the review, the following terms were searched for “(germination OR germinated OR bioactive) AND (brown OR white OR unpolished or polished) AND (antidiabetic OR glucose-lowering) AND rice.” Other publications were identified from references in publications found from search of the above databases. In addition to studies on metabolic outcomes relevant to type 2 diabetes (*in  vitro* data, animal experiments, and clinical studies) due to GBR, those that have reported on changes in bioactive constituents during the process of germination of BR were included. After excluding duplicate publications and reviewing abstracts, 12 publications on *in  vitro* and animal experiments were found to have reported on changes in metabolic indices relevant to type 2 diabetes due to GBR, while only 6 relevant clinical studies were found, out of which 2 were in Japanese language [46, 52]. Also, 27 studies were found to be relevant on germination studies of BR to produce GBR and are briefly reported too. 

## 3. Germination of Brown Rice

As can be recalled, several bioactive compounds present in BR, as shown in Table 1, are believed to be responsible for its functional effects. There have been reports of the compositional changes of BR bioactives, in an attempt to understand how their changes during germination affect the functional properties of GBR. In an earlier study, Tian et al. [18] had studied various phenolic compounds present in WR, BR, and GBR and found that all phenolic compounds were increased following germination of BR. Since phenolic compounds are reported to confer beneficial health effects, this may partly explain the better nutritive value of GBR over BR and WR. Findings by Jongjareonrak et al. [26] support those of Tian et al. [18]. They showed that germinating BR in tap water at 25°C significantly improved total phenolic content of BR and antioxidant activities as determined by DPPH and ABTS radical scavenging activity and ferric reducing antioxidant power (*P* < 0.05). Others have also reported similar findings [22, 23, 30, 35, 40]. Jiamyangyuen and Ooraikul [59] had reported also that GBR had lower amylose content consistent with reports in which activation of amylase enzyme was responsible for higher amounts of bioactives in GBR [60]. Musa et al. [36], Li et al. [41], and Liu et al. [42] also showed that amylose was significantly decreased in BR when subjected to germination. GABA is the most extensively studied GBR bioactive compound, and several studies have shown that it is increased during germination of BR [21, 22, 25, 27–30, 32, 33, 37, 39–41, 43, 59]. Similarly, Jayadeep and Malleshi [34] reported increased free sugars, insoluble fibre, and other bioactives following biotransformation of BR. Oryzanol, protein, fat, dietary sugar, and many more changes have been reported during germination of BR [19, 20, 24, 31, 34, 38]. Table 1 shows a summary of the main bioactive compounds found in BR and the compositional changes due to germination.

## 4. Studies on Mechanisms for Antidiabetic Effects of Germinated Brown Rice

A summary of studies on mechanisms of antidiabetic effects of GBR is shown in Table 2. Metabolic improvements due to GBR that may be beneficial in the management of type 2 diabetes include better glycemic control [13, 44–48, 51, 52, 55, 56], correction of dyslipidemia [46, 49, 51, 52, 54, 55, 57], amelioration of oxidative stress [40, 58], reduced type 1 tissue plasminogen activator inhibitor (PAI-1) [45, 56], and increased sodium potassium adenosine triphosphatase and homocysteine thiolactonase activities [50, 58]. Of the studies reported to date, only one has shown that GBR does not improve any metabolic indices in type 2 diabetes [44]. An analysis of these findings is given as follows. 

### 4.1. Preclinical Studies

Hagiwara et al. [45] reported that feeding streptozotocin-induced diabetic rats (fasting blood glucose of ≥300 mg/dL following injection of 65 mg/kg streptozotocin dissolved in 100 mmol/L sodium citrate buffer, pH 4.5) with GBR caused significantly improved glycemia after 7 weeks and reduced levels of PAI-1, triglycerides, and lipid peroxides. Seki et al. [48] used normal wistar rats to study glycemic and insulin indices over 2 h due to WR or GBR consumption and concluded that the lower glycemia and insulin response correlated well with amounts of dietary fibre in the GBR. In another study [56], feeding Otsuka Long-Evans Tokushima Fatty (OLEFT) rats, a model for type 2 diabetes in rats, with GBR or WR for 31 weeks showed that GBR could reduce levels of glycated hemoglobin A1c (HbA1c), tumor necrosis factor alpha, and PAI-1. GBR also improved adiponectin insufficiency more than WR did, suggesting that GBR could lessen inflammatory changes and insulin resistance. Accordingly, Shallan et al. [55] had reported that feeding with GBR for 5 weeks caused a significant reduction by up to 23%, 31%, and 45% in levels of glycemia, total cholesterol, and low-density lipoprotein (LDL) cholesterol, respectively, in diabetic albino rats. High-density lipoprotein (HDL) cholesterol was also reported to have increased by 45%. However, feeding with WR containing similarly low amylose content produced very similar findings in diabetic rats, prompting the conclusion that amylose content was the most important determinant of glycemia and lipid profile in GBR. Roohinejad et al. [54] however demonstrated that improvement in lipid profile of hypercholesterolemic rats (induced through feeding with a high fat diet for 4 weeks) after feeding with GBR for 6 weeks positively correlated with GABA content in GBR. Interestingly, feeding with GBR for 19 d was shown to not only improve glycemia and lipid profile, in support of the above findings, but it inhibited the growth of hepatoma cells as reported by Miura et al. [49] suggesting the wide enormous functional potentials of GBR. Furthermore, Mohd et al. [57] demonstrated that the chances of cardiovascular damage in rabbits following 10 weeks of consumption of a high-fat diet supplemented with GBR were significantly reduced (lower levels of total cholesterol, LDL, LDL/HDL, malondialdehyde and atherogenic index, reduced size of atherosclerotic plaque, and a higher level of HDL) compared to consumption of the high-fat diet alone. In this study, the improvements in these indices were however attributed to higher amounts of *γ*-oryzanol, tocopherol, and monounsaturated fatty acid content. 

As can be recalled, Tian et al. [18] and Kim et al. [35] reported that GBR grains had improved antioxidant capacity, which could potentially reduce oxidative stress. In addition, Usuki et al. [58] showed that reduction of oxidative stress by GBR may be due to its acylated steryl glycoside (ASG) content. Using a diabetic rat model (fasting blood glucose of ≥400 mg/dL following injection of 65 mg/kg streptozotocin dissolved in 100 mmol/L sodium citrate buffer, pH 4.5), they demonstrated that ASG could increase activities of sodium potassium adenosine triphosphatase and homocysteine thiolactonase enzymes, whose impairment is linked to oxidative stress in diabetic neuropathy and cardiovascular complications. Usuki et al. [50] further demonstrated that ASG in GBR improved several metabolic parameters (decreased levels of glycemia and LDL and increased levels of HDL, homocysteine thiolactonase activity, nerve conduction velocity, and serum insulin) in diabetic rats (fasting blood glucose of ≥400 mg/dL following injection of 65 mg/kg streptozotocin dissolved in 100 mmol/L sodium citrate buffer, pH 4.5) through induction of insulin-like growth factor. Increased amounts of serum, liver, and pancreatic insulin-like growth factor, a mediator of insulin action, suggested that ASG induced the production of the growth factor in diabetic rats, which was confirmed by findings from an *in vitro* model. Induction of insulin-like growth factor from insulin-secreting cells suggests that ASG could protect the cells from further damage in type 2 diabetes through reducing oxidative stress. Furthermore, Imam et al. [40] reported higher antioxidant potentials for GBR compared to WR and BR and demonstrated that GBR improved glycemia, total antioxidant status, and kidney hydroxyl radical scavenging capacity in type 2 diabetic rats (fasting blood glucose > 250 mg/dL in high-fat diet-induced obese rats, following injection of 35 mg/kg, streptozotocin dissolved in 100 mmol/L sodium citrate buffer, pH 4.5). They demonstrated that higher antioxidant potentials due to higher phenolic compounds and GABA content in GBR may have been responsible for the better antioxidant effects of GBR over WR and BR and also argued that nutrigenomic upregulation of superoxide dismutase gene may be involved in GBR's antioxidant effects.

### 4.2. Clinical Studies

Panlasigui and Thompson [13] had observed in a randomised crossover study involving 10 normal (3 males, 7 females; ages 24–50 with mean age of 33 ± 3.4 years; 100 ± 10% ideal weight except one subject who was 32% overweight and another who was 10% underweight) and 9 diabetic subjects (5 males, 4 females, age 45–64 with a mean age of 55 ± 2.9 years) that BR containing 50 g of available carbohydrate portion produced lower glycemic indices than a corresponding amount of WR 60 min after ingestion. Glycemic indices for BR and WR were 82.62 ± 14.62 and 94.00 ± 10.66 in normal subjects, and 56.00 ± 12.34 and 87.00 ± 11.40 in diabetic subjects, respectively. Furthermore, Noriega et al. [44] had reported that consumption of WR, BR, and GBR containing 50 g available carbohydrate portions by healthy subjects produced the same glycemic and insulin responses though findings by Ito et al. [47] contradicted this. In 2 separate studies to investigate the effects of GBR on glycemic and insulinemic responses, Ito et al. [47] used 19 (12 male, 7 females, age 23–41 years, body mass index 15.4–28.8 kg/m^2^) and 13 (5 males, 8 females, age 25–32, body mass index 15.4–25.6 kg/m^2^) normal human subjects, respectively, without any history of abnormal blood glucose readings within the previous year. In the study, GBR containing 50 g available carbohydrate portion produced better glycemic and insulin responses than corresponding amounts of BR and WR. Within 120 min of ingestion of test diets among 19 subjects (WR, BR, or GBR), GBR produced the lowest glycemic index (56.9 ± 2.9) when compared to BR (61.5 ± 4.7) or WR (79.5 ± 6.6). Also, when increasing concentrations of GBR were used in WR diet among 13 subjects, an exclusive GBR diet produced the lowest glycemic index (54.4 ± 5.1) compared to 2/3 GBR in WR diet (63.7 ± 5.3), 1/3 GBR in WR diet (67.4 ± 2.9), or an exclusive WR diet (74.6 ± 6.2). By and large, their data suggested that consumption of GBR may generate lower glycemic response and less chances of postprandial hyperglycemia-induced oxidative reaction in comparison to BR and WR. The data on glycemic responses due to GBR suggest that the glycemic index of GBR is lower than most commonly consumed foods, as shown in Table 3. 

Furthermore, Morita et al. [46] reported that a diet composed of GABA-rich GBR and WR did not produce significant changes in most metabolic indices in healthy individuals. Using 67 healthy volunteers (aged 71 ± 8), effects of WR and GBR + WR (1 : 1, w/w) were determined following consumption for 11–13 months. There was only significant decrease in HbA1c in the GBR + WR group, but no differences were noted in body mass index, blood pressure, serum lipids, and homeostasis model assessment of insulin resistance between the two groups. This could have been due to the presence of WR in the diet as demonstrated by Ito et al. [47] that improvements in metabolic indices caused by GBR are better produced with an exclusively GBR diet, and addition of WR reduces such benefits. In another study [51], 11 diabetic subjects (6 males, 5 females, age 27–72, body mass index 18.9–31.2 ± 3.4 kg/m^2^, fasting blood glucose > 110 mg/dL) were involved in a crossover design that lasted 14 weeks (6 weeks of feeding with either GBR or WR and a 2-week washout period in between). Subjects were maintained on their medications throughout the intervention (1 on insulin and 10 on oral hypoglycemics) and given 180 g of the respective rice diet 3 times a day. Between the initial and final interventions, WR did not produce significant changes in glucose and fructosamine levels after feeding for 6 weeks (from 147 ± 9 mg/dL to 150 ± 9 mg/dL and from 317.0 ± 10.1 *μ*mol/L to 315.2 ± 10.5 *μ*mol/L, resp.) unlike GBR which reduced both parameters significantly (from 153 ± 9 mg/dL to 135 ± 7 mg/dL and from 321.0 ± 10.7 *μ*mol/L to 303.0 ± 9.5 *μ*mol/L, resp.). Also, consumption of GBR significantly reduced total cholesterol and triglycerides and increased HDL cholesterol more than WR but had no effect on body mass index, serum proteins, or blood pressure. In a related study using 24 diabetic subjects, Hayakawa et al. [52] also observed that higher intake of GBR over 3 months did not have a remarkable effect on blood glucose but was associated with better HbA1c levels (from 6.40 ± 0.23% to 6.2 ± 0.19) and LDL-c/HDL-c ratio (from 2.03 ± 0.13 to 1.83 ± 0.12). Insulin levels and insulin resistance were also shown to have improved after 3 months of intervention. 

## 5. Discussion

Germination of BR is one way of increasing bioactive concentrations to enhance its functional effects as shown on Table 1. It is not yet clearly understood which bioactives are responsible for GBR's functional effects, but it is likely that several of the bioactive compounds contribute towards any observed effect. The dietary fibre in GBR is known to lower glycemic index through regulating the absorption of glucose in the intestines, as reported by Ou et al. [61] and supported by findings from Seki et al. [48]. Affectation of GABA receptors in pancreatic islets is partly responsible for decreased insulin release in type 2 diabetes [62], and GABA supplementation has been reported to increase insulin release [63]. This may explain reduction in blood glucose in diabetes caused by GABA [64]. In addition to glucose lowering, Nakagawa et al. [65] also reported that GABA had antioxidative effects. Oryzanol was reported by Nagasaka et al. [66] to prevent hypoadiponectinemia, which is implicated in reduced insulin sensitivity in diabetes [67]. In addition to oryzanol, GABA has also been reported to have a similar effect against hypoadiponectinemia [68]. ASG has been shown to regenerate sodium potassium adenosine triphosphatase and homocysteine thiolactonase enzymes with potential to reverse diabetic neuropathy and oxidative changes on biomolecules [50]. It has also been reported to improve the overall metabolic condition in diabetes as a result of induction of insulin-like growth factor-1 and reduced oxidative stress [58], which is a problem in type 2 diabetes. Several improvements in metabolic indices have also been directly linked to certain bioactive compounds in GBR [48, 54, 55, 57]. A wide range of other effects have also been reported for oryzanol and ferulic acid including antioxidative, antihyperglycemic, and hypocholesterolemic properties [69, 70]. The benefits from bioactives in GBR, as suggested earlier, may be explained by the concept of food synergy, proposed by Jacobs and Tapsell [71]. If so, through synergism GBR could on the long run be beneficial in managing type 2 diabetes through multiple mechanisms perhaps even better than some drugs as suggested by Brand-Miller et al. [72] that diets can have as much importance in management of type 2 diabetes as hypoglycemic drugs. Through potentiation of the concentration of bioactive compounds during germination of BR, the increased amounts of bioactives may synergistically interact to produce a better functional effect than any of the individual nutrients in GBR [71]. This suggests that the benefits from GABA, oryzanol, phenolics, and other bioactive compounds in GBR may be optimal when consumed as a whole food (GBR) compared to its individual bioactive compounds. The interaction of these bioactive compounds in GBR and the matrix in which they are held together by nature may be some of the factors that aid in their synergism. This way, overall functional effects are boosted when more of the bioactive compounds are present, which may explain why GBR produces better functional effects than BR. 

Preclinical findings from studies on mechanisms involved in antidiabetic effects of GBR suggest that GBR could improve metabolic indices in type 2 diabetes. From the studies reviewed, it can be deduced that GBR could improve a number of parameters that may be beneficial in management of type 2 diabetes. It is shown to reduce glycemia, insulin index, hypercholesterolemia, oxidative stress, HbA1c, PAI-1, and tumor necrosis factor alpha. It has also been shown to improve adiponectin insufficiency, total antioxidant status, and kidney hydroxyl radical scavenging capacity and also protect against neuropathy. Findings from clinical studies mirror some of those from preclinical data, though only very few have been reported making a conclusion difficult. Specifically, there are no long-term clinical studies that have documented long-term effects of GBR on metabolic indices in type 2 diabetes, and studies that have been reported were mostly done using healthy subjects. Though using normal subjects is still a limitation of most of the studies on antidiabetic effects of GBR, it may be argued that metabolic changes as a result of GBR could be similar in normal and diabetic subjects. The inclusion of the study (10 normal and 9 diabetic subjects) conducted using BR was to support the argument that even though BR is proven to be better than the commonly consumed WR as has been established, GBR is now proven to be superior to BR. Out of the 5 studies using GBR as test diet, 2 studies reported its effects on glycemic and insulin indices over 1-2 h, providing little information on its long-term effects on metabolic control in type 2 diabetes. Interestingly, of these 2 studies, 1 showed that GBR did not improve glycemic and insulin indices [44] though an apparent limitation of the study was that it was conducted using normal subjects. It is also likely that the rice type used did not have sufficient nutritional value to produce the expected metabolic changes. Aside from the 2 studies on effects of GBR on glycemic and insulin indices, the 3 short-term studies followed up only 102 subjects (35 diabetic and 67 normal subjects) for 14 weeks to 13 months. Though there are some indications that GBR improves metabolic indices in type 2 diabetes, it is hard to draw a conclusion based on only these clinical studies due to low population size. When using diabetic subjects, drug ingestion may be an important confounding factor to contend with. This may have limited long-term studies on effects of GBR on type 2 diabetes, though 1 study [51] in the current review maintained diabetic subjects on their medications throughout the intervention period. Put together, the findings from clinical studies suggest that GBR may reduce glycemia, hypercholesterolemia, fructosamine levels, and HbA1c, and improve insulin and insulin resistance. This suggests that GBR could improve symptomatology and prevent complications of type 2 diabetes since these factors are mostly implicated in both cases.

Insulin deficiency and/or insensitivity is the underlying cause of chronic sustained hyperglycemia in type 2 diabetes, which causes damage to several vital organs in the human body [73]. However, insulin's central role in intermediary metabolism results in metabolic perturbations affecting metabolism of all macromolecules. Because other derangements are secondary to hyperglycemia, most management modalities for type 2 diabetes are aimed at maintaining normoglycemia in order to prevent complications [73, 74]. Drugs, dietary, and lifestyle modifications aimed at ensuring antihyperglycemia and therefore remain at the core of managing the disease. In addition, high insulin index is implicated in promoting complications of type 2 diabetes [12, 75]. GBR's antihyperglycemic effects and lower insulin index suggest that the core problem in type 2 diabetes could be improved by GBR, thereby reducing the chances of other secondary perturbations and hyperglycemia-induced complications from arising. This in fact suggests that GBR could lessen the burden of type 2 diabetes. Accordingly, it is well known that adiponectin insufficiency affects insulin sensitivity and in some cases has even been reported to be a prelude to type 2 diabetes. Also, adiponectin insufficiency has been reported to play a role in the development of cardiovascular diseases [67, 76, 77]. On the other hand, there have been reports of improved insulin sensitivity in type 2 diabetes and a reduced risk of cardiovascular diseases with improved adiponectin levels. If GBR improved adiponectin concentration, this could suggest that such effect is one of the mechanisms by which GBR improves glycemic control in type 2 diabetes. Its effect on adiponectin insufficiency therefore suggests that GBR could potentially prevent cardiovascular diseases in addition to improving glycemic control. 

Cardiovascular diseases inflict the highest morbidity and mortality in type 2 diabetes, largely as a result of dyslipidemia [78, 79], which may be complicated by hypercoagulability as a result of abnormalities of PAI-1 [80, 81]. The fact that GBR has been reported to reverse dyslipidemia to some extent suggests that it could prevent or at least delay complications secondary to dyslipidemia, which account for a large chunk of the disability due to type 2 diabetes. PAI-1 has long been known to promote damage to cardiovascular and renal systems [82, 83], especially in type 2 diabetes, resulting in significant morbidity and mortality due to complications arising from such damage. Antithrombosis due to GBR could lower risk of damage to the vascular system especially of the cardiovascular and renal systems. Peripheral vascular disease as a result of thrombosis, which could result in amputations, may also be prevented in this way. 

Oxidative stress has been linked to the development of most chronic diseases [84, 85]. Specifically, in type 2 diabetes, it is thought to play a role in both pathogenesis and course of the disease [86, 87]. Type 2 diabetes creates a condition conducive for the promotion of oxidative stress, and in turn free radicals produced in excess mediate some of the harmful effects of hyperglycemia [88, 89], which manifest as complications of the disease. Ceriello et al. [90] had reported that even diet could promote oxidative stress in type 2 diabetes. It would therefore be imperative that any management modality for the disease takes this into account, considering that oxidative stress is already in excess in type 2 diabetes, and it is a known contributor towards complications of the disease. What is apparent from studies on GBR is that it could reduce oxidative stress in type 2 diabetes, and could therefore prevent or at least delay oxidative stress-related complications. Meal-generated oxidative stress as reported by Ceriello et al. [90] may also be less when GBR is consumed due to its lower glycemic index. Also, closely associated with oxidative stress are derangements in sodium potassium adenosine triphosphatase and homocysteine thiolactonase enzymes, which have been implicated in diabetic neuropathy [91, 92] and oxidative changes that affect the cardiovascular and other systems, respectively [93, 94]. Improvements in activities of these enzymes by GBR could suggest that complications of diabetic neuropathy and cardiovascular disease arising from respective enzyme derangements may be prevented or even reversed. Reduction in oxidative stress could reduce chances of complications in type 2 diabetes [5]. 

Finally, as suggested from the antidiabetic effects of GBR presented in this paper (Table 2), GBR may offer several metabolic improvements in type 2 diabetes and even prevent complications. In addition, hypertension is a problem for many diabetics [95] and appropriate management of blood pressure has been shown to reduce cardiovascular complications in this disorder [96]. Therefore, antihypertensive effects of GBR [53], may provide additional protection from cardiovascular complications. These effects could have a profound impact on the overall burden of type 2 diabetes. It may also be argued that GBR could potentially lessen the burden of type 2 diabetes in low- and middle-income countries of Asia and Africa where rice is the staple diet, by preventing major complications of the disease. Also, other diseases in which some of the metabolic factors earlier mentioned are known to contribute significantly may be prevented or ameliorated by GBR. In particular, the link between oxidative stress and many chronic diseases is widely acknowledged, and GBR could affect the course of such oxidative stress-related diseases. Already, there have been reports of successful incorporation of GBR into food products like bread, and consumer willingness to purchase the products is encouraging [97–99]. Development of such products may bring closer the benefits of GBR to a wide range of people as suggested by Patil and Khan [17]. However, loss of bioactive compounds in products developed from GBR may be a concern. Kong and Lee [100] had reported that addition of GBR to noodles increased the GABA content of the noodles, suggesting that even after processing and cooking, bioactive compounds and their benefits may still be retained. Charoenthaikij et al. [101] also reported minimal change to properties of GBR after incorporating up to 50% of its flour into bread product. 

Eventhough what has been reported on GBR suggests it may improve metabolic indices in type 2 diabetes, future studies are still needed to determine long-term effects of GBR and its bioactive compounds using larger populations of diabetic subjects and to examine effects of different processing methods and product development on bioactive compounds. The results of these studies may help to establish GBR as an antidiabetic functional food that will benefit rice-consuming populations and will also increase understanding on the optimum conditions for minimizing loss of bioactives during processing and product development.

## 6. Conclusions

Recent evidence suggests that diet is an important factor in worsening of type 2 diabetes; hence, it can be used for control of this disease by diabetic patients. *In vitro* data, animal experiments, and clinical studies suggest GBR to have strong potentials of an antidiabetic functional food that could provide a steady supply of bioactive compounds, for maintaining health and preventing complications in type 2 diabetes. Populations where rice is the staple diet may derive the most benefit from such effects if they choose to consume GBR instead of WR. However, further studies including long-term clinical trials on diabetic subjects using different GBR products are needed to establish the full potential of GBR in the management of type 2 diabetes.

## Figures and Tables

**Table 1:** Summary of compositional changes of the main bioactive compounds in brown rice during germination.

Reference	Main bioactive changes*
Tian et al. (2004) [18]	↑Phenolics, ↑antioxidant activity
Britz et al. (2007) [19]	↑Alpha-tocopherol, ↑alpha-tocotrienol, ↑gamma-oryzanol, ↓gamma-tocopherol, ↓gamma-tocotrienol
Lee et al. (2007) [20]	↑Protein, ↑fat, ↑dietary fibre, and ↑free sugars
Komatsuzaki et al. (2007) [21]	↑GABA
Li et al. (2008) [22]	↑Total phenolic compounds, ↑total dietary fiber, ↑GABA, and ↑phytate
Sawaddiwong et al. (2008) [23]	↑Phenolic content, ↑antioxidant activity
Usuki et al. (2008) [24]	↑Acylated steryl glycoside
Banchuen et al. (2009) [25]	↑GABA, ↑protein, ↑fat, ↑dietary fibre, ↑free sugar, ↑ferulic acid, *↔*oryzanol, and ↓phytate
Jongjareonrak et al. (2009) [26]	↑Phenolics, ↑antioxidant activity
Banchuen et al. (2010) [27]	↑GABA, ↑ferulic acid, ↓phytate, and *↔*oryzanol
Charoenthaikij et al. (2010) [28]	↑GABA
Jannoey et al. (2010) [29]	↑GABA
Maisont and Narkrugsa (2010) [30]	↑GABA, ↑dietary fibre, ↑total phenolics content, ↑antioxidant capacity, ↓amylose, ↓fat, and ↓protein
Moongngarm and Saetung (2010) [31]	↑Crude proteins, ↓B-vitamins, and ↓phytic acid
Oh et al. (2010) [32]	↑Dietary fibre, ↑GABA, ↑gamma-oryzanol, *↔*fatty acid, *↔*protein, and *↔*ash
Watchararparpaiboon et al. (2010) [33]	↑GABA, ↑protein, ↑lipids, ↑thiamine, and ↑phytate
Jayadeep and Malleshi (2011) [34]	↑Free sugars, ↑soluble fibre, ↑tocotrienol, ↓insoluble fibre, ↓fat, ↓tocopherol, ↓total antioxidant activity, and *↔*gamma-oryzanol
Kim et al. (2011) [35]	↑Antioxidant activity
Musa et al. (2011) [36]	↓Amylose, *↔*starch granule
Roohinejad et al. (2011) [37]	↑GABA
Xu et al. (2012) [38]	↑Reducing sugars, ↑ash, and ↓amylose
Karladee and Suriyong (2012) [39]	↑GABA
Imam et al. (2012) [40]	↑GABA, ↑total phenolic content, and ↑antioxidant capacity
Li et al. (2012) [41]	↓Amylose, ↑reducing sugars, ↑GABA, and ↑dietary fibre
Liu et al. (2012) [42]	↓Amylose, *↔*starch granule
Songtip et al. (2012) [43]	↑Reducing sugars, ↑GABA

↑: increased, ↓: decreased, and *↔*: not changed. GABA: gamma-aminobutyric acid. *Bioactive compounds present in brown rice, which undergo changes during the process of germination to confer germinated brown rice with its enhanced functional effects.

**Table 2:** Summary of findings on mechanisms for antidiabetic properties of germinated brown rice.

Reference	Study type/comments	Summary of main changes in metabolic indices
Noriega et al. (1993) [44]	Assessed glycemic and insulin indices in healthy subjects using 50 g available carbohydrate portions	*↔*Blood glucose, *↔*insulin response

Hagiwara et al. (2004) [45]	Assessed changes in various metabolic indices in diabetic rats (fasting blood glucose of >300 mg/dL) induced through i.p. injection of 65 mg/kg body weight of STZ dissolved in 100 mmol/L sodium citrate buffer, pH 4.5	↓Blood glucose, ↓PAI-1, and ↓lipid peroxidation

Morita et al. (2004) [46]	Assessed effects of WR and GBR + WR (1 : 1, w/w) in 67 healthy volunteers (aged 71 ± 8) over 11–13 months	↓HbA1c, *↔*BMI, *↔*blood pressure, *↔*lipid profile, *↔*kidney and liver functions, *↔*blood glucose, and *↔*insulin response

Ito et al. (2005) [47]	Assessed glycemic and insulin responses over 120 min in 2 studies using 19 (12 male, 7 females, age 23–41 years, body mass index 15.4–28.8 kg/m^2^) and 13 (5 males, 8 females, age 25–32, body mass index 15.4–25.6 kg/m^2^) healthy subjects. Intervention compared 50 g available carbohydrate portion of GBR, BR, and WR	↓Blood glucose, ↓insulin response

Seki et al. (2005) [48]	Assessed glycemic and insulin indices of WR and GBR in normal Wistar rats over 120 min	↓Blood glucose, ↓insulin response

Miura et al. (2006) [49]	Assessed effect of GBR on hypercholesterolemia in hepatoma bearing rats for 19 d	↓Total cholesterol

Usuki et al. (2007) [50]	Assessed effect of GBR on diabetic rats (fasting blood glucose of >400 mg/dL) induced through i.p. injection of 65 mg/kg body weight of STZ dissolved in 100 mmol/L sodium citrate buffer, pH 4.5, and effect of acylated steryl glycoside on *in vitro* cultures of rat cells	↓Glucose, ↑nerve conduction velocity, and ↑HTase

Hsu et al. (2008) [51]	11 diabetic subjects (6 males, 5 females, age 27–72, body mass index 18.9–31.2 ± 3.4 kg/m^2^, fasting blood glucose > 110 mg/dL) in a crossover design for 14 weeks (6 weeks of feeding with either GBR or WR and a 2-week washout period in between) were used to assess effects of 180 g test diets 3 times daily; subjects were maintained on their medications (1 on insulin, 10 on oral hypoglycemics)	↓Blood glucose, ↓fructosamine, ↓total cholesterol, and ↓triglycerides

Hayakawa et al. (2009) [52]	Assessed various metabolic indices in 24 diabetic subjects on WR or GBR over 3 months	↓HbA1c, ↓insulin response, ↓HOMA-IR, ↓LDL cholesterol, ↑HDL cholesterol, and *↔*blood glucose

Ebizuka et al. (2010) [53]	Assessed serum lipids and blood pressure on spontaneously hypertensive rats on control or 40% GBR diet for 8 weeks	↓Total cholesterol, ↓blood pressure

Roohinejad et al. (2010) [54]	Assessed serum lipids in diet-induced hypercholesterolemic rats on control diet, 24 h germinated BR diet, or 48 h germinated BR diet for 6 weeks	↓Total cholesterol, ↓LDL cholesterol

Shallan et a. (2010) [55]	Assessed changes in metabolic indices in diabetic albino rats on control or GBR diets for 5 weeks	↓Weight, ↓glucose, and ↓cholesterol

Torimitsu et al. (2010) [56]	OLEFT rats (model of type 2 diabetes) were used as diabetic animals and LETO rats as nondiabetic controls to assess effects of GBR and control diet on various metabolic indices for 30 weeks	↓Blood glucose, ↓TNF, ↓PAI-1, and ↑adiponectin

Mohd et al. (2011) [57]	Assessed various metablic indices in diet-induced hypercholesterolemic rabbits on GBR, BR, or WR diets for 10 weeks	↓Total cholesterol, ↓LDL cholesterol, ↓atherogenic index, ↓MDA, and ↑HDL cholesterol

Usuki et al. (2011) [58]	Assessed effects of GBR on metabolic indices of diabetic rats (fasting blood glucose of >400 mg/dL) induced through i.p. injection of 65 mg/kg body weight of STZ dissolved in 100 mmol/L sodium citrate buffer, pH 4.5 and effect of ASG on *in vitro* cultures using INS-1 cells	↓Glucose, ↓LDL cholesterol, ↑nerve conduction velocity, ↑HTase, ↑IGF-1 (↓oxidative stress), and ↑insulin secretion

Imam et al. (2012) [40]	Assessed effects of WR, BR, and GBR on glycemia and antioxidant status of diabetic rats (fasting blood glucose of >250 mg/dL) induced through i.p. injection of 35 mg/kg body weight of STZ dissolved in 100 mmol/L sodium citrate buffer, pH 4.5, in high-fat diet-induced obese rats	↓Glucose, ↑total antioxidant status, ↑hydroxyl radical scavenging capacities of liver and kidneys, and ↑expression of superoxide dismutase gene

↑: increased, ↓: decreased, and *↔*: not changed. BMI: body mass index; HDL: high-density lipoprotein; HTase: homocysteine thiolactonase; HOMA-IR: homeostasis model assessment-estimated insulin-resistance; IGF: insulin-like growth factor; INS-1: insulin-secreting cell lines; i.p.: intraperitoneal; LDL: low-density lipoprotein; LETO: Long-Evans Tokushima Otsuka; MDA: malondialdehyde; OLEFT: Otsuka Long-Evans Tokushima Fatty; PAI-1: type 1 tissue plasminogen activator inhibitor; STZ: streptozotocin; TNF: tumor necrosis factor.

**Table 3:** Glycemic indices of white rice (WR), brown rice (BR), and germinated brown rice (GBR) in comparison to other commonly consumed foods.

Reference	Glycemic index of food
Panlasigui and Thompson (2006) [13]	Study 1: in normal subjects WR 94.00 ± 10.66 BR 82.62 ± 14.62 Study 2: in diabetic subjects WR 87.00 ± 11.40 BR 56.00 ± 12.34

Ito et al. (2005) [47]	Study 1: in normal subjects WR 79.50 ± 6.60 BR 61.50 ± 4.70 GBR 56.90 ± 2.90 Study 2: in normal subjects WR 74.60 ± 6.20 2/3WR + 1/3GBR 67.40 ± 2.90 1/3WR + 2/3GBR 63.70 ± 5.30 GBR 54.40 ± 5.10

Harvard Health Publications*	White wheat flour bread 71 Whole wheat bread, average 71 Coca Cola, average 63 Cornflakes, average 93 Oatmeal, average 55 Couscous, average 65 Puffed wheat, average 80

*Glycemic index and glycemic load for 100+ foods, from http://www.health.harvard.edu/newsweek/Glycemic_index_and_/glycemic_load_for_100_foods.htm.

## Abbreviations

ASG: Acylated steryl glycoside
BR: Brown rice
GABA: *γ*-Aminobutyric acid
GBR: Germinated brown rice
HbA1c: Glycated hemoglobin
HDL: High-density lipoprotein
LDL: Low-density lipoprotein
PAI: Type 1 tissue plasminogen activator inhibitor
WR: White rice.
